# How Do Cryo-Milling and Lyophilization Affect the Properties of Solid Dispersions with Etodolac?

**DOI:** 10.3390/pharmaceutics17111379

**Published:** 2025-10-24

**Authors:** Anna Czajkowska-Kośnik, Radosław A. Wach, Eliza Wolska, Katarzyna Winnicka

**Affiliations:** 1 Department of Pharmaceutical Technology, Medical University of Białystok, Mickiewicza 2c, 15-222 Białystok, Poland; katarzyna.winnicka@umb.edu.pl; 2 Department of Institute of Applied Radiation Chemistry, Faculty of Chemistry, Łódź University of Technology, Wróblewskiego 15, 93-590 Łódź, Poland; radoslaw.wach@p.lodz.pl; 3 Department of Pharmaceutical Technology, Medical University of Gdańsk, Hallera 107, 80-416 Gdańsk, Poland; eliza.wolska@gumed.edu.pl

**Keywords:** etodolac, solid dispersions, cryo-milling, lyophilization, drug solubility, DSC, FTIR

## Abstract

**Background:** Solid dispersions (SDs) of etodolac (ETD), a poorly water-soluble drug model, were developed to enhance its solubility and dissolution rate by employing various preparation methods and hydrophilic or amphiphilic polymers. **Methods:** Polyvinylpyrrolidone-poly(vinyl acetate) copolymers (PVP/VA), hydroxypropyl methylcellulose (HPMC) and poloxamer were used as carriers, while cryo-milling and lyophilization were utilized as routine methods to SDs preparation. Obtained SDs were characterized by drug content, solubility, dissolution rate and moisture content. The physical structure of SDs was estimated via scanning electron microscopy (SEM), whereas differential scanning calorimetry (DSC) and Fourier transform infrared spectroscopy (FTIR) were employed to assess the potential drug-carrier interactions. **Results:** SD formulations demonstrated enhanced solubility of ETD in aqueous media, including water and buffers (pH 5.5 and 7.4). DSC analysis confirmed that PVP/VA and poloxamer ensured better ETD dissolution and protection against recrystallization. Furthermore, FTIR indicated the formation of hydrogen bonds between ETD and polymer, particularly in lyophilized dispersions. **Conclusions:** The optimized SD formulation for ETD contained PVP/VA and/or poloxamer as carriers and was obtained via lyophilization. This SD formulation exhibited the most favorable properties, enhanced the solubility and dissolution of ETD in aqueous media and effectively reduced its crystallinity.

## 1. Introduction

**Figure 1:**
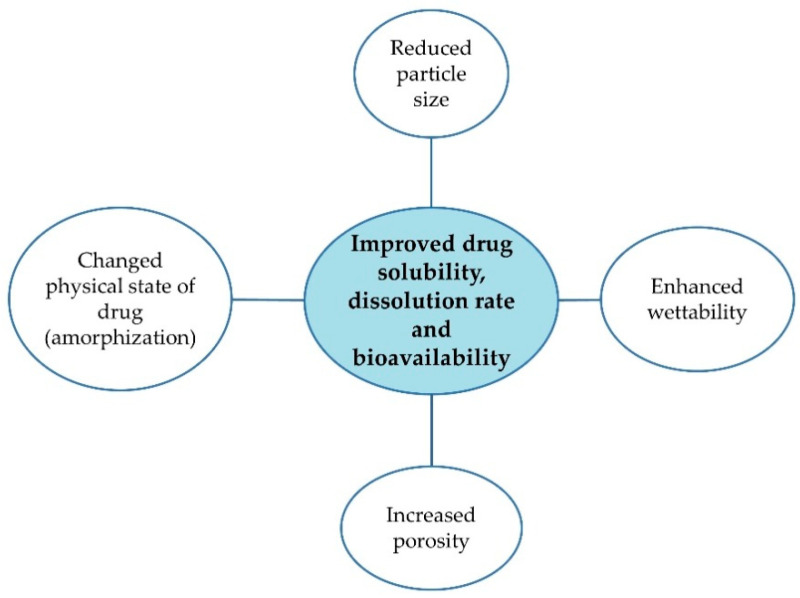
Merits of SDs [8,9,10].

Poor water solubility is one of the main reasons for inadequate drug bioavailability and unsuccessful formulation development steps. To improve solubility, different strategies are employed and various techniques are studied, such as micronization, salt formation, complexation, use of surfactants and solubilizers [1,2,3]. Solid dispersions (SDs) constitute one of the approaches to enhance drug solubility, especially promising for drugs belonging to a Biopharmaceutics Classification System (BCS) class II, characterized by low solubility and high permeability [4,5,6,7]. SDs are described as a mixture of at least two components, a hydrophobic drug and a hydrophilic matrix. They can lead to particle size reduction, increased wettability and porosity, reduced agglomeration and drug amorphization (Figure 1) [8,9,10].

Etodolac (ETD) is chemically described as 1,8-diethyl-l,3,4,9-tetrahydropyrano-[3,4-b] indole-1-acetic acid and represents the selective COX-II (cyclooxygenase) inhibitor non-steroidal anti-inflammatory drug (NSAID) [11,12]. It has analgesic, anti-inflammatory and antipyretic activity and can be used to treat acute pain, rheumatoid arthritis and osteoarthritis. Due to its low solubility (0.016 mg/mL) [12] and some drawbacks of conventional oral therapy (gastrointestinal disturbances, frequent dosing), the alternative ways to drug administration—buccal, topical and nasal route—are reviewed. Some studies present utilization of ETD in topical dosage form, e.g., liposomes, solid lipid nanoparticles, nanostructured lipid carriers, cubosomes, ethosomes, nanosuspensions, nanoemulsions, film-forming spray and gels. Madhavi et al. [13] found that liposome and ethosome gels containing ETD exhibited better pharmacokinetic and pharmacodynamic profiles (prolonged half-life, increased mean residence time in the body and greater reduction in edema in a rat model) compared to conventional industrial gel. Increased ETD skin permeability was also achieved by Patel et al. [14], who incorporated the drug into a gel based on solid lipid nanoparticles. Other researchers who combined topical preparations (creams, gels) with innovative drug carriers (nanoemulsions, nanosuspensions, nanostructured lipid carriers, cubosomes) also demonstrated that these systems are promising formulation strategies for ETD and then highlighted the need to continue the exploration of new, advanced carriers for ETD [15,16,17,18,19,20,21,22,23].

The carriers used in SDs present crystalline form (e.g., urea, sugars), representing the first generation of SDs, or in amorphous state (e.g., PVP, HPMC), representing the second generation of SDs. SDs containing other excipients, such as an additional polymer or surfactant, are known as the third generation of SDs [1,24,25,26]. Many methods are available for SDs preparation, including solvent evaporation, spray drying, hot-melt extrusion (HME), ball milling (grinding), lyophilization (freeze-drying) and electrospinning. Among the commonly used technologies, spray-drying and HME are considered as standard methods for SDs preparation. Spray-drying provides good molecular dispersion but possesses the risk of exposure to organic solvents, while HME requires high processing temperatures, which can lead to decomposition of excipients and API [27,28,29,30]. Given these challenges and exploring alternative solutions, other preparation techniques such as cryo-milling and lyophilization seem to be attracting approaches. In our study, we chose these two methods to prepare optimized SDs. Cry-omilling, a modified form of conventional ball milling (at room temperature condition), is a solvent-free, low-temperature and simple method to obtain SDs. This method has been widely employed in pharmaceutical research to enhance drug solubility [31,32,33,34]. In comparison to ball milling, during cryo-milling, the ingredients are frozen using liquid nitrogen to minimize the risk of thermal degradation of the drug and other components [32]. During milling, drug amorphization probably occurs as a result of milling-induced disorders, generated by major mechanical perturbations (reduction in particle size, polymorphic transformations, partial or complete amorphization) [32,35,36].

Lyophilization is a process in which water and organic solvent at the first stage is frozen, then removed from the sample by sublimation process [37,38]. An important merit of lyophilization is that drugs are not exposed to high temperatures. In this method, poor water-soluble drugs are preferred because they weakly bond to solvents and are more easily transformed in to dried form. In the literature, shorter dissolution times for lyophilized products have been reported compared to solid forms obtained by other methods (e.g., solvent evaporation) [8,39,40,41,42].

The prepared SDs can be examined using different analytical and instrumental methods. Commonly studied parameters of SDs are solubility, dissolution rate, physical state of drug (amorphous or crystalline) and morphology. The list of methods for SDs characterization is presented in Table 1. In our study, one method from each type was chosen to assess the properties of designed SDs.

In this work, PVP/VA, HPMC and poloxamer were selected as ETD polymer carriers, based on previous studies [44]. PVP/VA (copovidone), known by its trade name as Kollidon VA64, is a copolymer containing six parts of vinylpyrrolidone and four parts of vinylacetate. It is characterized as an amorphous polymer that is soluble in hydrophilic solvents, commonly used as a binder and film-forming agent. In contrast to PVP (polyvinylpyrrolidone), PVP/VA is less hygroscopic, exhibits a glass transition temperature of approximately 100 °C and degrades at high temperature (above 230 °C) [7,45]. Hydroxypropyl methylcellulose (HPMC) is one of the most widely used cellulose ethers in solid dispersions and other pharmaceutical formulations. It is characterized as a non-ionic, water-soluble and non-pH-responsive compound. HPMC as a non-toxic, biocompatible and biodegradable ingredient is mainly used as dispersing and viscosity-modifying agent [46,47]. Even though this polymer lacks very strong hydrogen bond donor and acceptor groups, HPMC is used in many marketed drugs with SDs (tablet or capsule dosage form) [48,49,50]. In the structure of HPMC, both the hydrophilic (hydroxy) and lipophilic (ether) groups are identified, so it proves high drug–polymer miscibility and/or solubility [7,48,51].

In this work, the possibility of using SDs as an approach to improve the solubility of a poorly water-soluble drug model was evaluated. Etodolac (ETD) belongs to a Biopharmaceutics Classification System Class II and is characterized by low aqueous solubility. Polymers with varying physicochemical properties, including poly(vinylpyrrolidone-co-vinyl acetate) (PVP/VA), hydroxypropyl methylcellulose (HPMC) and poloxamer were tested as carriers. The choice of amorphous carriers—HPMC and PVP/VA—was based on the results from previous studies [31]. Cryo-milling and lyophilization were utilized as methods to obtain SDs formulations. SDs were characterized by drug content, solubility, dissolution rate, morphology, interactions and physical state of ETD. This study aims to assess the effects of the preparation methods of SDs—cryo-milling and lyophilization—on improving ETD solubility. This thesis is a continuation of prior work [44], which focused on SDs with ETD obtained by ball milling process.

## 2. Materials and Methods

### 2.1. Materials

Etodolac (ETD) was obtained from Xi’an Health Biochemical Technology Co. (Xi’an, China). Kollidon VA 64 (polyvinylpyrrolidone-poly(vinyl acetale) copolymers, PVP/VA) was supplied from BASF (Burgbernheim, Germany), Pharmacoat 606 (hydroxypropyl methylcellulose, HPMC) from Shin-EtsuChemical Co. (Niigata, Japan) and poloxamer 407 (poly(ethyleneglycol)-block-poly(propyleneglycol)-block-poly(ethyleneglycol)from Sigma Aldrich (Steinheim, Germany). Potassium dihydrogen phosphate and sodium acetate were provided by Chempur (Piekary Sląskie, Poland), ethanol 96% and acetic acid from POCH (Gliwice, Poland) and acetonitrile from J.T. Baker (Deventer, Holland). Water for HPLC analysis was obtained by a Milli-Q Reagent Water System (Millipore, Billerica, MA, USA).

### 2.2. Analytical Method

Concentrations of ETD during drug content, solubility and dissolution studies were measured by high-performance liquid chromatography (HPLC) with modification [52], using Agilent Technologies 1260 Infinity equipment (Agilent, Waldbronn, Germany) and Eclipse XDB C18 column (5 µm, 4.6 × 250 mm; Agilent Technologies, Boblingen, Germany). For this purpose, the analysis was conducted as follows: wavelength 225 nm, column temperature 25 °C and an isocratic flow rate (1 mL/min) of the mobile phase (acetonitrile/0.02 M phosphate buffer pH 6.0 in 50:50 *v/v* ratio). The standard calibration curve was linear in the range from 0.5 to 7.5 µg/mL (R^2^ = 0.999), while the limit of detection was 0.15 μg/mL. The intraday and interday precisions for concentration 5 µg/mL were between 0.34 and2.93%, respectively.

### 2.3. Production of SDs Containing ETD by Cryo-Milling and Lyophilization Method

Cryo-milling and lyophilization were selected as laboratory-scale techniques for preparing SDs with ETD. These methods are accessible and cost-effective, they do not require specialized equipment. Importantly, both techniques permit processing under mild conditions without exposure to high temperatures, which may be advantage for API and other excipients (protection from thermal stress) [31,33,34]. Different formulations of solid dispersions with etodolac (ETD-SDs) containing drug and polymers (HPMC, PVP/VA, poloxamer) in a 1:2 weight ratio were prepared using the cryo-milling process. In brief, ETD and polymer were weighted and mixed using a multi-rotator (Multi RS-60, Riga, Latvia) for 10 min at 100 rpm. The prepared physical mixtures (PMs) were then put in steel jars containing 3 steel balls (10 mm diameter) (Supplementary Materials, Table S1). Immediately prior to the milling, the jars were immersed inliquid nitrogen (−196 °C) for 5 min. The cryo-milling was run for 30 or 60 min using Retsch GmbH MM 400 ball mill (Haan, Germany). To prevent overheating, 2 or 4 cycles of 15 min with repeated freezing in liquid nitrogen were performed. The parameters of milling: time (30/60 min) and frequency (20 Hz) were established in the earlier studies [44]. K0 and K00 formulations consisted of pure ETD and were prepared to compare the effect of the polymers. All formulations were stored in Eppendorf tubes in a desiccator in the presence of anhydrous calcium chloride. The composition of ETD–SD prepared by cryo-milling is presented in Table 2.

**Figure 2:**
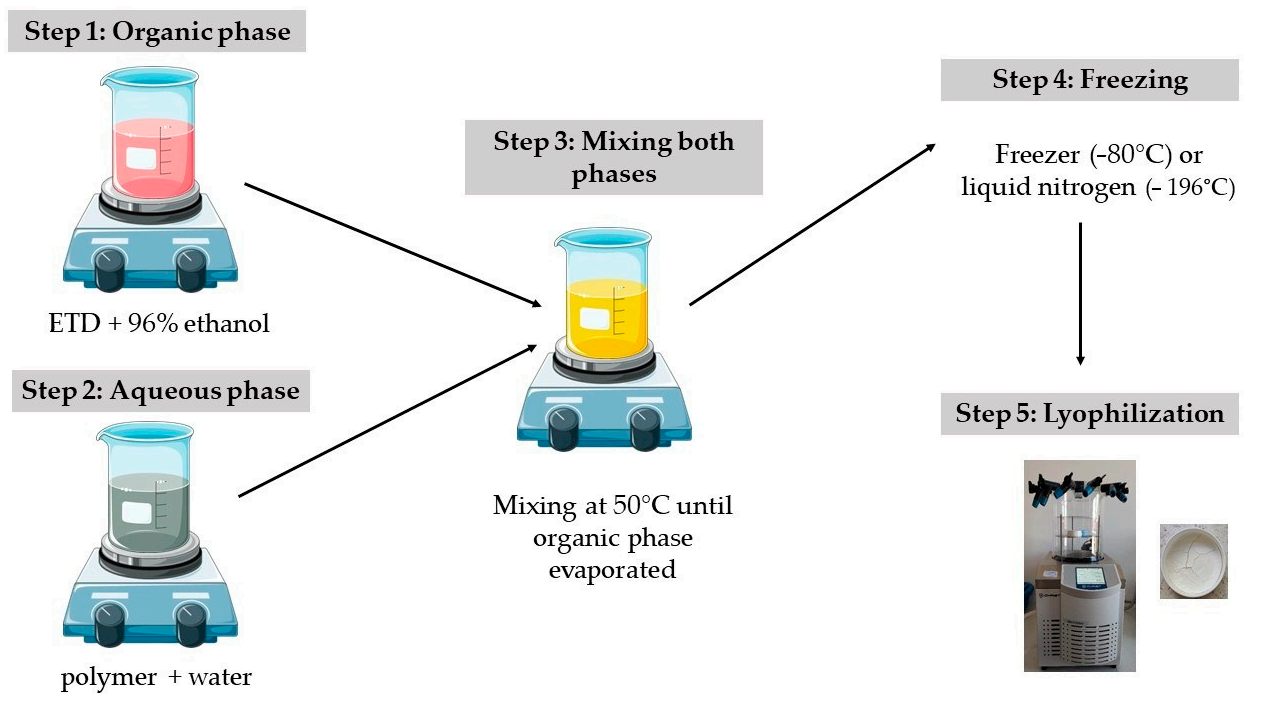
Preparation method of ETD-SDs by lyophilization.

In the second method to obtain SDs, a lyophilizer Alpha 2-4 LSC Basic (Martin Christ, Osterodeam Harz, Germany) was used (Figure 2). In the first step, aqueous dispersions of the drug and polymer were prepared. For this, 1 g of etodolac was dissolved in 96% ethanol, and the carrier was solubilized in 20 g of water. Then, these two phases were blended using a magnetic stirrer (400 rpm), mixed and heated (50 °C ± 2 °C) until the organic solvent evaporated (the volume of solution was reduced to half of the initial state). Liquid formulations were put in plastic boxes and frozen with liquid nitrogen (−196 °C, 5 min) or in the freezer (−80 °C, 24 h). Then, frozen formulations were lyophilized for 24 h under pressure 0.05 mBar (−48 °C), with additional drying step for 2 h (0.005 mBar). Lyophilized SDs were stored in a desiccator with anhydrous calcium chloride. The compositions of ETD–SD prepared by lyophilization are presented in Table 2.

### 2.4. Drug Content Assay

SDs were tested for ETD content by HPLC method, as described in Section 2.2. Samples were prepared by dissolving SDs in acetonitrile, shaking for 24 h (250 rpm) at 25 °C ± 1 °C, and then by filtering through a 0.2 µm acetate cellulose (CA) membrane, diluted with mobile phase and analyzed. The experiment was repeated 3 times.

### 2.5. Morphological Assessment

The surface morphology of ETD, pure carriers and SDs was characterized by scanning electron microscopy (SEM) Phenom Pro (Phenom World Thermo Fisher, Eindhoven, The Netherlands). Samples were mounted on aluminum stubs by carbon adhesive tape, then covered with a thin layer of gold and observed under magnifications of 1000×.

### 2.6. Thermal Analysis

Thermal analysis was performed using a differential scanning calorimeter (DSC Q200, TA Instruments, New Castle, DE, USA). The instrument was calibrated for both temperature and enthalpy using indium with melting temperature and heat of fusion of 156.6 °C and 28.57 J·g^−1^, respectively. A sample weighing 3–5 mg was placed in an aluminum pan. Samples were heated from 10 °C to 200 °C at a rate of 10 °C·min^−1^ under nitrogen flow 50 mL·min^−1^. The thermal stability of ETD and selected formulations was examined using thermogravimetric analysis (TGA, Mettler Toledo, Greifensee, Switzerland). Samples of c.a. 4–6 mg in aluminum pan were heated to 600 °C under argon atmosphere with a heating rate of 20 °C·min^−1^.

### 2.7. Fourier Transform Infrared Spectroscopy

The formulations (solid samples) were blended with potassium bromide in 1% concentration, compressed with a laboratory press and examined by Fourier Transform Infrared Spectroscopy (FTIR) with Thermo Scientific Nicolet IS10 (Waltham, MA, USA), by scanning in the wavelength range of 4000–500 cm^−1^ with a resolution of 1 cm^−1^ and 64 scan number.

### 2.8. Moisture Content and Hygroscopicity Tests

The moisture of SDs was measured thermogravimetrically using a weighing dryer (Radwag Ma 50.R, Radom, Poland). The experiment was carried out at 60 °C, until a constant weight. The sample weight loss and moisture percentage were recorded. Three assays were run for each sample.

A known amount of SDs formulations were placed on the glass plates and transferred to the climate chamber (Memmert HCP 2, Memmert GmbH & Co., Schwabacg, Germany). The hygroscopicity experiment was programmed for water sorption under different temperature and relative humidity conditions (25 °C/60% RH, 30 °C/65% RH, 40 °C/75% RH). Each step was carried out for 48 h, after which the mass changes in the samples were evaluated. Visual observation was also performed.

### 2.9. Solubility Studies and Dissolution Testing

The solubility of ETD in various media was performed, using a shake-flask method. The excess amount of SDs were added to water, phosphate buffer pH 7.4 or acetate buffer pH 5.5, and then the vials were transferred to the water bath for 24 h (rotated at 250 rpm) at 25 °C ± 1 °C. In the next step, the suspensions were centrifuged (4000 rpm, 10 min) and filtered through a 0.2 µm CA membrane filter (Alfachem, Poznan, Poland). The concentration of ETD was assessed by HPLC. The study was performed in triplicate.

The dissolution studies of ETD from SDs were carried out in a USP dissolution Apparatus II (Erweka DT 600D, Heusenstamm, Germany). Each SDs formulation (equivalent to 50 mg of ETD) was placed in the vessel with 250 mL of medium (phosphate buffer pH 7.4 or acetate buffer pH 5.5). The temperature was kept at 37 °C ± 0.5 °C, while the stirring of paddles was set at 100 rpm. Samples of acceptor fluid (2 mL) were withdrawn at predetermined time intervals (and replaced with fresh media), filtered through 0.2 µm CA filters and then determined by the HPLC method. References—unprocessed ETD, pure cryo-milling or lyophilized ETD (K0 and L0) were applied as controls. The dissolution rate experiment was conducted in triplicate. The summary of the results is presented on a graph, illustrating the relationship of percentage drug release vs. time. To compare the dissolution profiles of pure ETD, cryo-milled ETD (K0), lyophilized ETD (L0) and SDs, the dissolution efficiency (DE, %) and mean dissolution time (MDT, min) were calculated [53].

### 2.10. Statistical Analysis

The results were presented as arithmetic means ± standard deviations (S.D.) and analyzed using the StatSoft Statistica 13.0 software (StatSoft, Kraków, Poland). The normality of the variable distribution was checked with the Shapiro-Wilk test. Data characterized by normality of distribution was assessed by Tukey test, while those without normality were evaluated by the Kruskal-Wallis test. Measurements were considered significant at *p* < 0.05.

An AI tool (ChatGPT5, OpenAI) was used for language editing to improve readability of the manuscript.

## 3. Results and Discussion

### 3.1. Preparation of SDs with ETD, Drug Content

The lyophilization method is one of the ways used to prepare amorphous SDs containing poorly water-soluble drugs, where the final lyophilized product is characterized by high porosity, which affects drug solubility and long-term stability [54,55]. During lyophilization, L1-F and L1-N formulations were not tested. They formed very viscous mixtures with an incompletely dissolved carrier (HPMC) and therefore the homogenous solutions could not be obtained. The remaining liquid dispersions were frozen using a freezer (L0, L1-F to L5-F) or liquid nitrogen (L00, L1-N to L5-N). L0 and L00 formulations included only pure ETD. L3-F, L3-N, L4-F and L4-N contained PVP/VA, while poloxamer was used as an addition in L4-F and L4-N. HPMC was incorporated in L2-F, L2-N and L1-N formulations, while L2-F and L2-N additionally contained poloxamer. In L5-F and L5-N formulations, ETD and only one carrier (poloxamer) were applied.

**Figure 3:**
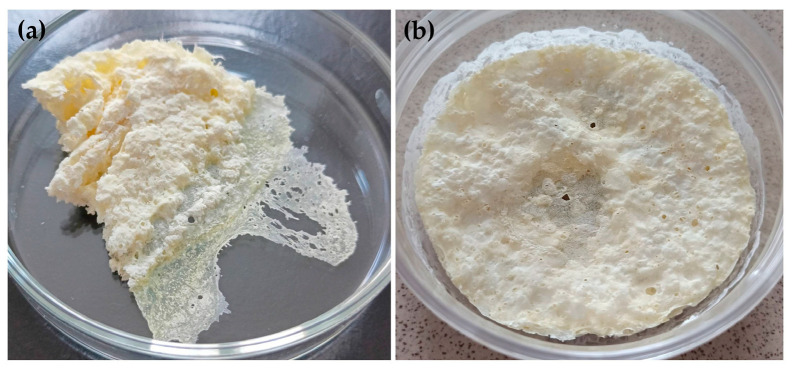
Visual appearance of (**a**) L2-F and (**b**) L2-N formulations.

**Figure 4:**
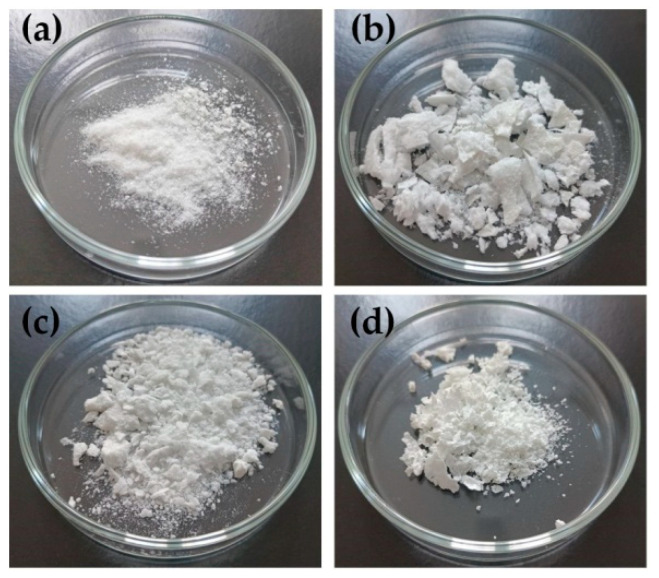
Visual appearance of (**a**) L0, (**b**) L3-F, (**c**) L4-F and (**d**) L5-F.

The prepared SDs were visually evaluated. After lyophilization, L0, L00, L3-F, L3-N, L4-F, L4-N, L5-F and L5-N formulations existed as a white, crushed disk. L2-F and L2-N (containing HPMC) formed a white-yellow solid mass that was impossible to crush. They were eliminated from further testing (Figure 3). In the next stage, L0, L00, L3-F, L3-N, L4-F, L4-N, L5-F and L5-F were crushed in a mortar and passed through a 0.5 mm sieve. Due to the specific form of the tested material (lyophilized disk crushed to powder), particle size measurements were not conducted. After the crushing step, SDs formulations existed as dull, flowing powders, while L0 and L00 (pure ETD)as a shiny, flowing material. The selected formulations are depicted in Figure 4.

**Figure 5:**
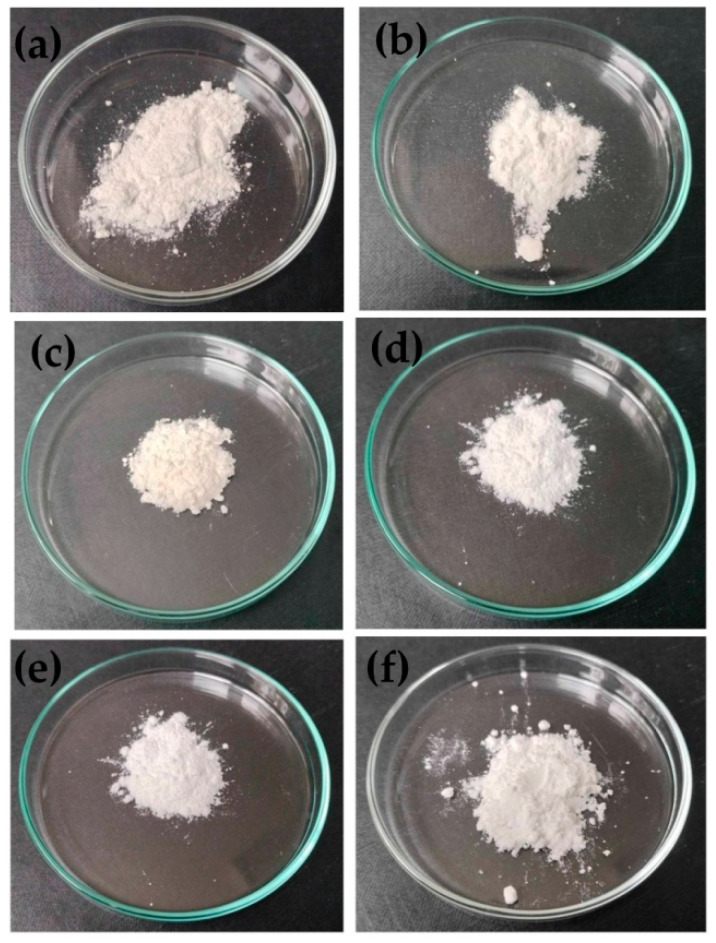
Visual appearance of (**a**) K00, (**b**) K2-60, (**c**) K1-60, (**d**) K3-30, (**e**) K4-30 and (**f**) K5-30.

Introducing cryo-milling as a modification of conventional ball milling ensures protection against drug overheating. In this study, SDs with PVP/VA, HPMC and poloxamer were prepared by freezing in liquid nitrogen and then milling in a ball mill, for 30 or 60 min. The obtained formulations were characterized as white, flowing powders (Figure 5). After milling, only K1-30 and K1-60 formed hard surface on jars’ walls, which required scraping. Finally, each formulation was passed through a 0.5 mm sieve.

The mean ETD content in the prepared formulations ranged from 95.6 to108.5% for lyophilized SDs and from 94.2 to 106.7% for cryo-milled SDs. These results indicated the usability of applied carriers and methods for ETD-SDs preparation.

### 3.2. Thermal Evaluation

**Figure 6:**
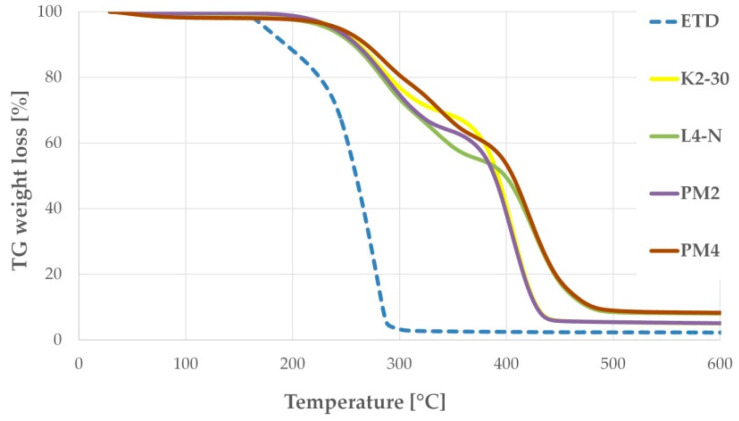
TG curves of selected formulations: unprocessed ETD, PMs (PM2: ETD + HPMC + poloxamer and PM4: ETD + PVP/VA + poloxamer) and SDs (K2-30 and L4-N).

Thermogravimetry (TG) is an important analytical method that enables us to observe the thermal stability of excipients, weight loss due to desolvation and thermal degradation. In this study, pure ETD, selected PMs and SDs were analyzed by thermogravimetric tool to assess their thermal stability, prior DSC examination. Figure 6 presents no loss of ETD mass up to about 158 °C, which indicates its stability over the melting point, and followed by thermal decomposition progressing to about 290 °C [12,56]. In TGA spectra of PMs and SDs, a shift in the ETD mass loss was recorded at temperature above 170 °C. Thermal degradation of ETD in PMs and SDs occurred slowly. In formulations with HPMC and poloxamer (PM2, K2-30), the substances lost entire mass at below 450 °C, whereas formulations with PVP/VA and poloxamer (PM4, L4-N) kept decomposing to about 480 °C. This study noticeably confirmed delayed decomposition of ETD when it was bounded with carriers, both in the form of PM and SDs. This fact was described also by Gupta R.D. and Raghav N. [56], who studied the drug delivery system of ETD and nanocrystalline cellulose obtained by the grinding method and showed that the bounded drug exhibits higher thermal stability.

**Figure 7:**
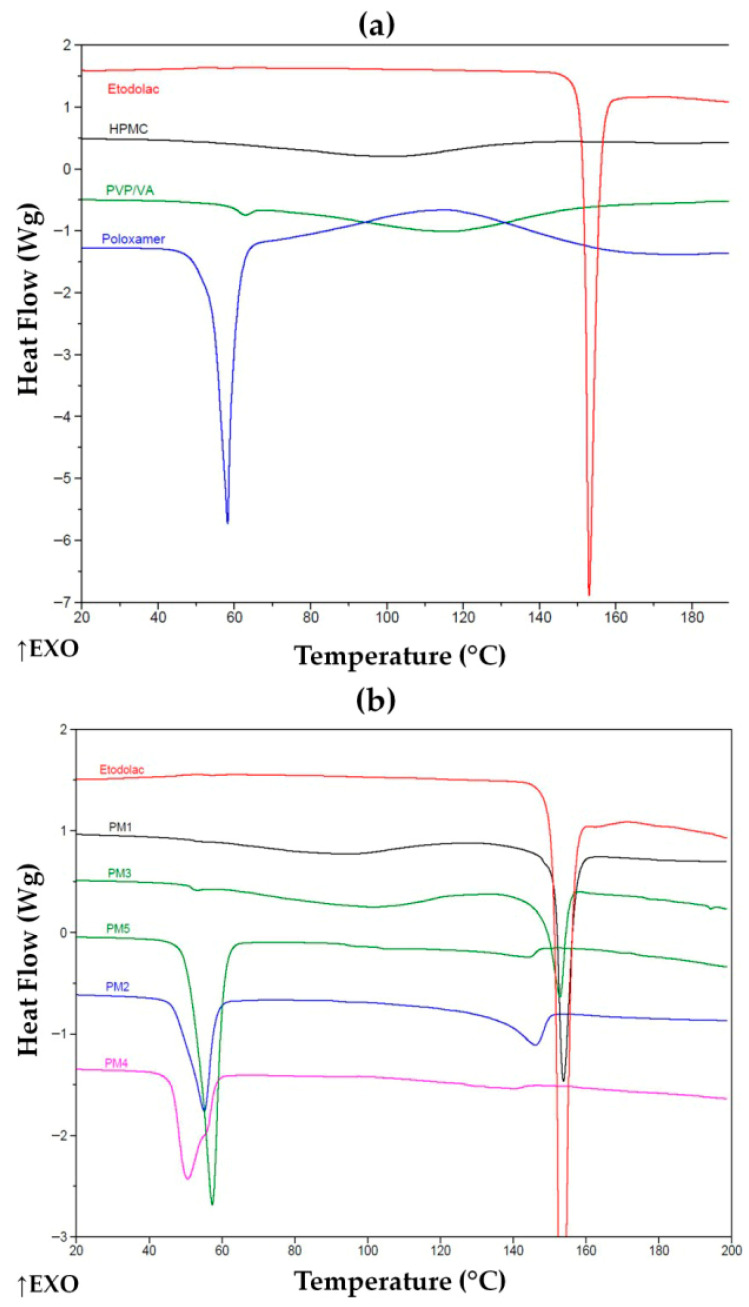
DSC thermograms of (**a**) unprocessed ETD and carriers and (**b**) unprocessed ETD and PMs. ↑EXO indicates an exothermic direction (heat release).

The DSC study was performed up to 200 °C in order to fully reveal melting of ETD crystallites and other transitions and keep the buffer before a decomposition of the components. The DSC thermograms of ETD, carrier components of HPMC, PVP/VA, poloxamer and PMs are presented in Figure 7. The DSC thermogram of pure ETD demonstrated a sharp endothermic peak with its maximum at 152–153 °C. This is characteristic of ETD melting, indicating the crystalline state of the pure drug. It is important to mention that a broad endotherm upon the first heating in some samples (with HPMC and PVP/VA) was attributed to the water loss of highly hydrophilic polymers, which was indicated in TGA thermograms as well. Water loss—a characteristic of PVP/VA copolymer—is depicted in the Supplementary Materials (Figure S1). Moreover, a glass transition in the copolymer was detected during the second heating at about 108.7 °C, which was approximate to value for pure homopolymers of PVP/VA (107.53 °C) [57]. To understand the possible interactions of the drug with the carrier components in the solid state, PMs were examined 24 h after their preparation by gentle mixing of fine powders (Figure 7b). The drug existed in its crystalline form in the HPMC (PM1) and PVP/VA (PM3) mixtures, with enthalpy of fusion (_Δ_H) of c.a. 53 ± 7 and 32 ± 6 J/g. Both mixtures were of ETD:polymer 1:2 ratio, but the melting effect of the drug was slightly different. When comparing the thermal effect of the crystals melt in ETD with an actual sample (determined as about 140 ± 11 J/g) and considering the composition, it is evident that the crystalline part of the drug was not affected by HPMC, but some interactions of ETD with PVP/VA reduced the crystalline part of the drug by about one-third. A pronounced effect of trituration was observed for poloxamer carrier, for which no crystallinity of the drug was detected. Indeed, a shift to lower temperature and the reduction in the thermal effect of poloxamer melting from about 134 ± 11 J/g for pure polymer, to c.a. 89 ± 15, 43 ± 4 and 48 ± 7 J/g for PM5 (only poloxamer), PM2 (HPMC and poloxamer) and PM4 (PVP/VA and poloxamer) revealed that this amphiphilic polymer effectively dissolved the drug. This dissolution occurred upon solid contact during 24 h storage rather than after its melting during heating in the DCS experiment, as no melting or dissolution was revealed in the thermogram. Slightly higher enthalpy of melting of poloxamer being in combination with PVP/VA than that with HPMC also evidences that the vinyl copolymer interacted with ETD, causing poloxamer remained less affected. Thermograms of PM2 and PM4 show changes inthe characteristic sharp peak of poloxamer. Its melting point represented by distinct endothermic peak at 58 °C has shifted [57,58]. These results indicate the interaction between ETD (poloxamer) and the solubilization of the drug in melted polymer.

**Figure 8:**
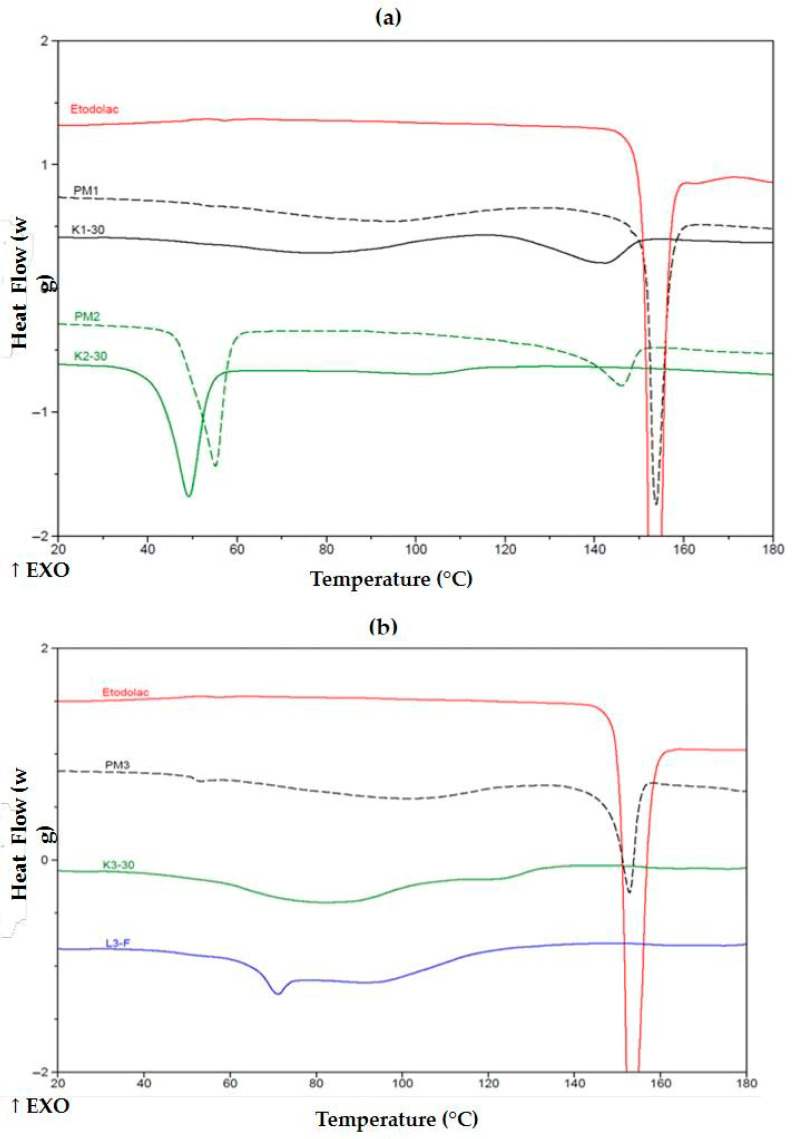
DSC thermograms of (**a**) unprocessed ETD, PMs (PM1, PM2) and SDs (K1-30, K2-30) and (**b**) ETD, PM3, K3-30 and L3-F. ↑EXO indicates an exothermic direction (heat release).

Thermograms of K1-30 exhibited no exothermic peak of ETD (Figure 8a), indicating that HPMC can effectively disperse upon cryo-milling and hinder further crystallization of the drug. Poloxamer alone or in combination with HPMC binds the drug that prevented its crystallization in either PMs (PM2, PM5) or upon cryo-milling (K2-30, K5-30). The latter revealed a lower melting temperature, undoubtedly indicating interactions formed prior to heating during the experiment. One may consider that the thermal effect initiating at about 140 °C for PM2 is melting some ETD crystals. This effect was found in all PMs with poloxamer, including PM2, PM4 and PM5. SDs containing PVP/VA, lyophilized K3-30 and cryo-milled L3-F were characterized by the absence of ETD melting peak (Figure 8b), indicating drug dissolution and that the copolymer prevented its recrystallization in both processes.

**Figure 9:**
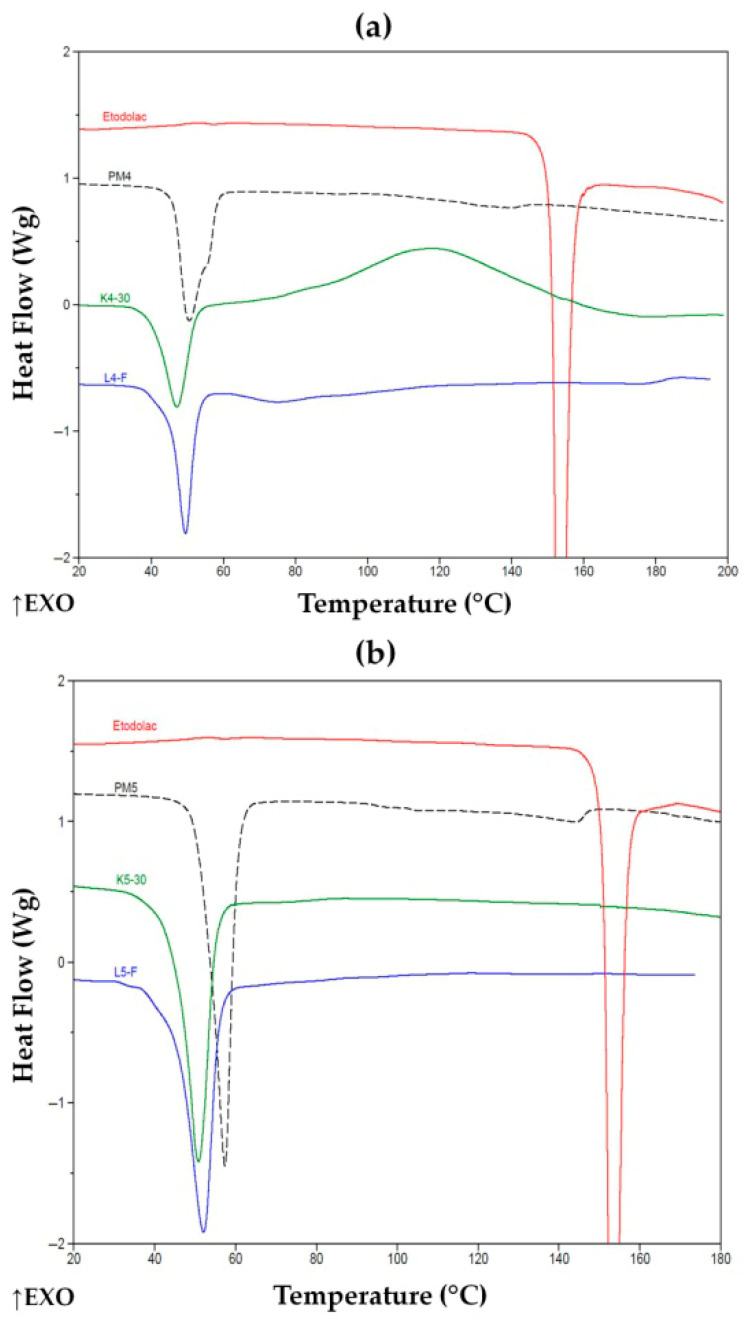
DSC thermograms of (**a**) ETD, PM4, K4-30 and L4-F and (**b**) ETD, PM5, K5-30 and L5-F. ↑EXO indicates an exothermic direction (heat release).

In Figure 9, PMs reveal no evident crystalline phase of the drug; however, as mentioned above, the thermal effect starting about 140 °C, indicated its existence in some extent, and drug dissolution in the melted poloxamer. Cryo-milled K5-30 and lyophilized L5-F formulations demonstrated no thermal effect, lower melting temperature of poloxamer and reduced its heat of fusion, both to about 80 ± 5 J/g in comparison with its PMs. This evidenced effectiveness to prevent crystallization of ETD. Similarly, poloxamer with PVP/VA formulations after cryo-milling or lyophilization reduced poloxamer melting temperature (slightly, but in PM4 a shoulder peak can be discriminated at c.a. 55 °C) and melting energy to about 38 ± 3 and 38 ± 5 J/g, respectively. These specific changes in melting behavior of the ETD and the carrier in formulations obtained by cryo-milling and lyophilization, revealed in DSC thermograms (Figure 8 and Figure 9), confirmed that designed SDs may release drug in a controlled way.

### 3.3. Morphological Assessment

**Figure 10:**
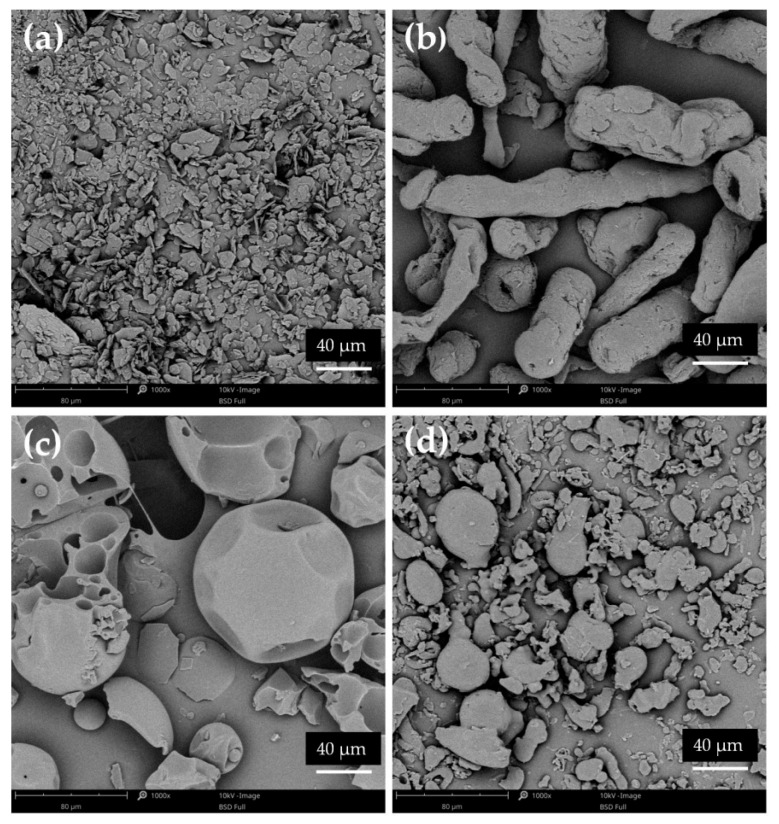
SEM images of pure (**a**) ETD, (**b**) HPMC, (**c**) PVP/VA and (**d**) poloxamer (magnification 1000×).

SEM analysis was performed for unprocessed ETD, carriers, cryo-milled and lyophilized ETD and SDs obtained by both methods. As shown in Figure 10, ETD existed as different-size particles (most of them below 40 µm), characterized by flat surfaces with sharp edges. HPMC was characterized as large, elongated fragments (“worm-like”), PVP/VA as big spherical particles with smooth surface and pores, while poloxamer appeared in variable shapes, mainly as spherical particles with smooth surfaces [58,59,60,61,62,63].

**Figure 11:**
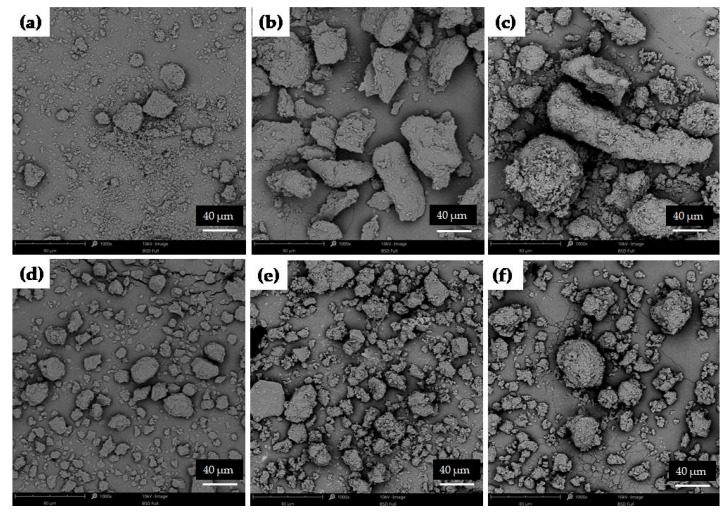
SEM images of cryo-milled ETD: (**a**) K0, (**b**) ETD–SD with HMPC (K1-60), (**c**) ETD–SD with HPMC and poloxamer (K2-30), (**d**) ETD–SD with PVP/VA (K3-30), (**e**) ETD–SD with PVP/VA and poloxamer (K4-30) and (**f**) ETD–SD with poloxamer (K5-30) (magnification 1000×).

The different compositions of SDs (various polymers) affected the varied morphology of obtained particles. Figure 11a presents that the cryo-milling process reduced the ETD particles; however, the formation of ETD aggregates was observed. SDs with HPMC possessed the characteristic structure of this carrier (Figure 11b,c). SEM analysis of SDs with poloxamer demonstrated a spherical morphology, which is characteristic of this carrier. Only for PVP/VA, its typical morphology was not visible on SEM scans (Figure 11d,e). The reduction in particles size of SDs formulations was noted in all images.

**Figure 12:**
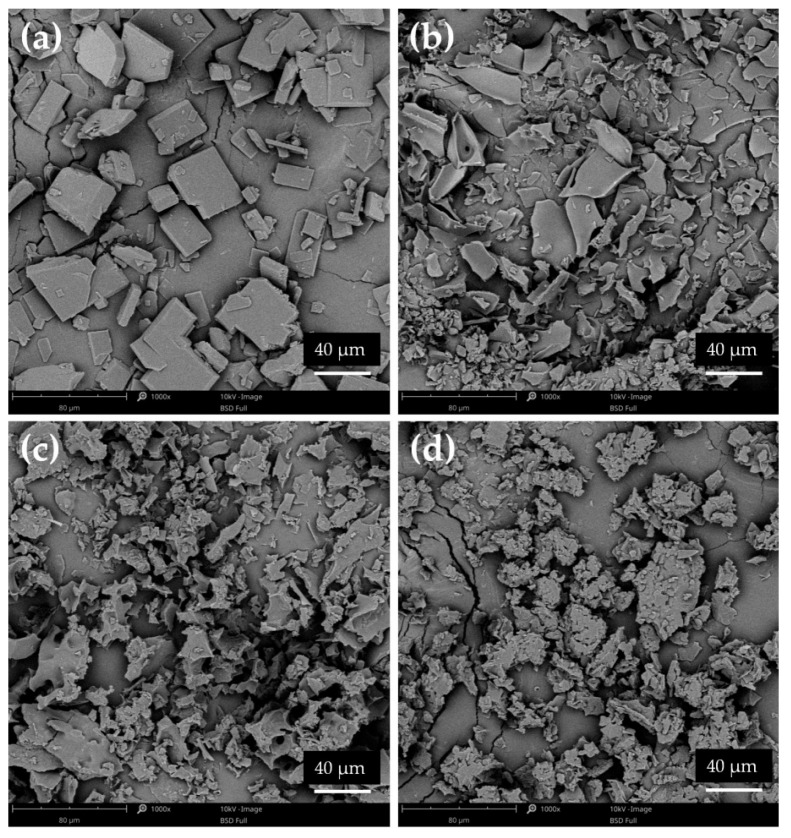
SEM images of lyophilized ETD: (**a**) L00, (**b**) ETD–SD with PVP/VA (L3-N), (**c**) ETD–SD with PVP/VA and poloxamer (L4-N) and (**d**) ETD–SD with poloxamer (L5-N) (magnification 1000×).

The specific structure of lyophilized formulations was a result of the lyophilization process, after which the products existed as a flat, smooth disk, which was later crushed and ground in a mortar. As shown in Figure 12a, the lyophilized ETD occurred as different-sized flat pieces. Similar images were obtained for other SDs. It was noted that the change in the morphology of the tested formulations—a characteristic structure of the polymers—was lost and a reduction in the sharp edges of ETD was observed. It can be concluded that the lyophilization method enabled the dispersion of ETD in polymers molecules and reduction ETD crystallinity, while cryo-milling process probably facilitated the formation of drug-carrier mixtures and/or dissolution of the drug in the polymer matrix (the structure of drug and polymers was mostly retained).

### 3.4. FTIR Study

**Figure 13:**
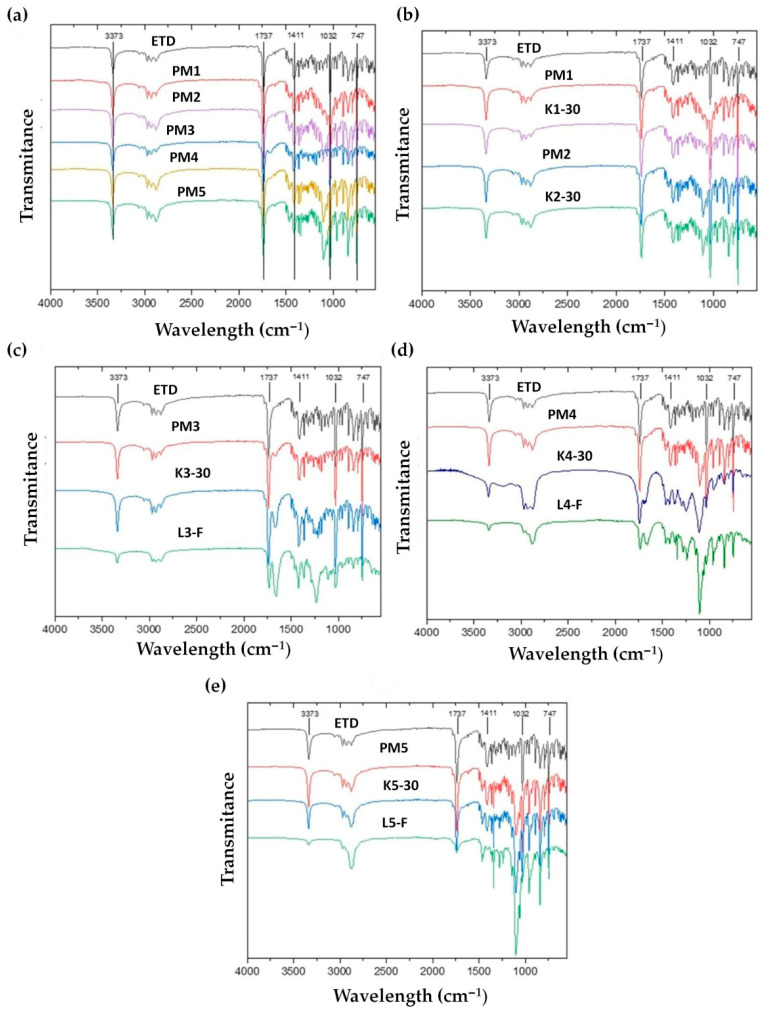
FTIR spectra of unprocessed (**a**) ETD and PMs, (**b**) unprocessed ETD, PM1, PM2, K1-30 and K2-30, (**c**) unprocessed ETD, PM3, K3-30 and L3-F, (**d**) unprocessed ETD, PM4, K4-30 and L4-F and (**e**) unprocessed ETD, PM5, K5-30 and L5-F.

Fourier-transform infrared spectroscopy (FTIR) analysis was conducted to identify the main bands of compounds and to indicate potential interactions between ETD and the carriers. FTIR spectra of unprocessed ETD, PMs and SDs are compiled in Figure 13. The most intense bands of the crystalline ETD were recorded in the FTIR spectra at about 3373 cm^−1^ (OH group of carboxylate moiety), 1737 cm^−1^ (C=O stretching), 1411 cm^−1^ (-CH_3_ asymmetric deformation), 1032 cm^−1^ (-CO stretching) and 747 cm^−1^ (-NH wagging mode) [64]. In the FTIR spectra of PMs (Figure 13a) and of cryo-milled SDs (K1-30, K2-30, K3-30, K4-30, K5-30), the characteristics signals for ETD were observed and no changes were noted. This indicates that there are no interactions between drug and carriers. However, the OH stretching vibration of ETD at 3373 cm^−1^ showed a decrease in intensity in lyophilized SDs (Figure 13c–e), which could indicate the participation of this group in the complexation process (hydrogen bonding). Singh J.K. et al. [63] examined the ETD-phospholipid complex, obtained by the solvent evaporation method, while Gadade D.D. et al. [65] analyzed the co-crystals of ETD and salicylic acid, prepared by the grinding method. Their solid dispersions—cocrystals and phospholipid complex, the same as in our study, exhibited a decrease in intensity of the OH peak. Moreover, in our research for characteristic peaks of ETD at 1737 cm^−1^, 1032 cm^−1^ and 747 cm^−1^, a decrease in their intensity was observed. The decrease in frequency of peaks indicated the formation of new hydrogen bonds and confirmed the interaction between ETD and carriers in SDs obtained by lyophilization. These effects were not observed in SDs prepared by cryo-milling technique.

In the lyophilization process, the drug and polymer are dissolved in solvents, then frozen and dried by sublimation. During solvent removal, drug and carrier molecules are in a molecularly dispersed state. Because they are initially in solution, they can form specific interactions (e.g., hydrogen bonding). As a result, increased drug–polymer bonding can stabilize the amorphous form of drug, prevent recrystallization and improve its solubility [24,66,67,68]. Cryo-milling is a mechanical size-reduction process performed at low temperatures. The drug and polymer are not dissolved, and this process relies on mechanical energy (leading to the fractured of solid particles, reduction in particles size and partial or complete amorphization) rather than molecular mixing. During cryo-milling, the drug and polymer often remain in a separate solid state and the formation of new hydrogen bonds is limited [36,69].

### 3.5. Moisture Content and Hygroscopicity Tests

**Figure 14:**
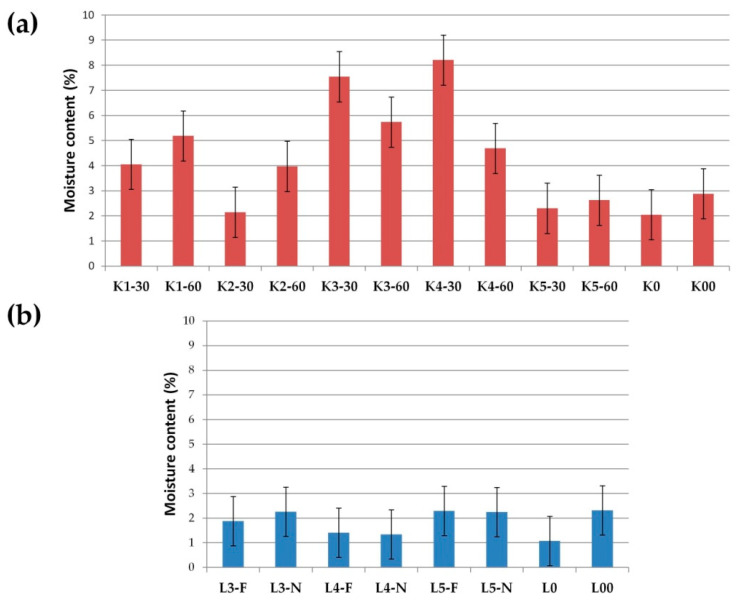
Moisture content (%) in (**a**) cryo-milled and (**b**) lyophillized formulations.

Prepared SDs were assessed by moisture content and hygroscopicity, as described in Section 2.8. It was noticeable that lyophilized SDs were characterized by lower humidity (Figure 14). The moisture content in the lyophilized SDs ranged from 1.08 to 2.32%, while in the cryo-milled SDs ranged from 2.05 to 8.21%. However, it should be noted that the moisture content in all SDs was at a low level (below 10%), and the lower humidity of lyophillized SDs related to the type of SDs preparation method. During the lyophilization method, at first, water in the formulations is frozen, then it is converted to steam in a sublimation reaction [37,70]. Additionally, in our study, an extra drying step (2 h) was applied, which resulted in a lower moisture content in the final products.

**Figure 15:**
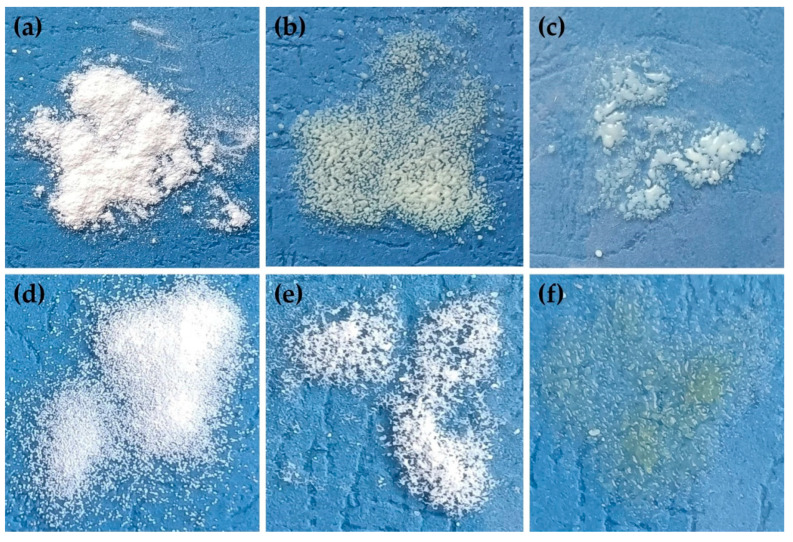
Visual observation of (**a**) K1-30, (**b**) K2-30, (**c**) K3-60, (**d**) L0, (**e**) L3-N and (**f**) L4-F, during hygroscopicity study (40 °C/75% RH).

Water sorption in pharmaceutical formulations could lead to changes in the properties of the drug (e.g., recrystallization) or other excipients or affect the quality of the drug formulation. Evaluating the sorption of water is also important to determine the storage conditions of formulation if it requires special conditions (e.g., hermetic package, moisture-absorbing agent) [71,72]. During the hygroscopicity test, all SDs were exposed to different conditions of temperature and humidity (25 °C/60% RH, 30 °C/65% RH and 40 °C/75% RH). Each step was carried out for 48 h, and the mass change in samples and visual observation were performed. For all cryo-milled and lyophillized SDs, low water sorption (less than 0.1%) was observed, while visual observation provided significant information. Cryo-milled SD formulations (K2-30, K2-60 and K4-30 to K5-60) exhibited signs of over-melting during storage at 30 °C (65% RH) and 40°C (75% RH). Additionally, some samples (K2-30, K2-60) underwent a noticeable color change from white to yellow, indicating potential physical or chemical instability under elevated temperature conditions. Similar changes were observed for lyophilized SDs such as L4-F, L4-N, L5-F and L5-N (SDs’ formulations with poloxamer). The melting point of poloxamer 407 is in the range from 52 °C to 57 °C; the experimental temperature was near these values, which could be the reason for the changes in SDs structure [73,74]. K3-30 and K3-60 formulations (with PVP/VA) melted at 40 °C in 75% RH, while unprocessed ETD, cryo-milled ETD (K0, K00) and lyophilized ETD (L0, L00), K1-30 and K1-60 (HPMC), L3-F and L3-N (PVP/VA) remained unchanged under the tested conditions. It can be concluded that the optimal storage conditions for SDs are ambient temperature (about 25 °C) and 60% humidity. Representative images from the hygroscopicity study are presented in Figure 15.

### 3.6. Solubility and Dissolution Rate

The solubilities of ETD in cryo-milled SDs (Table 3) were in the ranges of 0.10–0.16 mg/mL in water, 0.87–1.37 mg/mL in acetate buffer pH 5.5 and 1.91–2.44 mg/mL in phosphate buffer pH 7.4. Compared with pure ETD and pure milled ETD (K0, K00), a significant improvement in its solubility was observed for all prepared SDs. The maximum ETD solubility in water and buffer pH 5.5 exhibited K2-60 (HPMC + poloxamer), while in buffer pH 7.4-K5-60 (poloxamer). In SDs containing various carriers–HPMC, PVP/VA and poloxamer–no significant differences (*p* > 0.05) were observed between the ETD solubilities. For each formulation, the best ETD solubility was noted in phosphate buffer (above 2 mg/mL for most SDs), which relates to the chemical properties (acidic nature) of the drug. ETD is characterized by a pKa value of 4.65, which means that at higher pH (pH > pKa), it presents mainly in ionized form and shows better solubility [12,75]. Also, for lyophilized SDs, the ETD solubility was improved compared to pure and lyophilized ETD (L0, L00) (Table 4). In this case, ETD dissolved in water, buffer pH 5.5. and buffer pH 7.4 in the ranges of 0.20–0.43 mg/mL, 1.02–1.69 mg/mL and 2.10–3.08 mg/mL, respectively. The highest ETD solubility in water showed L4-F (PVP/VA, poloxamer), and in both buffers, it showedL3-F (PVP/VA). Similarly to the cryo-milled SDs, the lyophilized SDs showed no statistically significant differences (*p* > 0.05) in ETD solubility across different carriers. The highest ETD solubility was observed in phosphate buffer at pH 7.4. Comparing the two methods and their effect on improvement of ETD solubility, a greater impact was seen for lyophilized SDs, especially solubility of ETD in water, which was four times greater for lyophilized SDs (formulations with PVP/VA and poloxamer) than for cryo-milled SDs.

**Figure 16:**
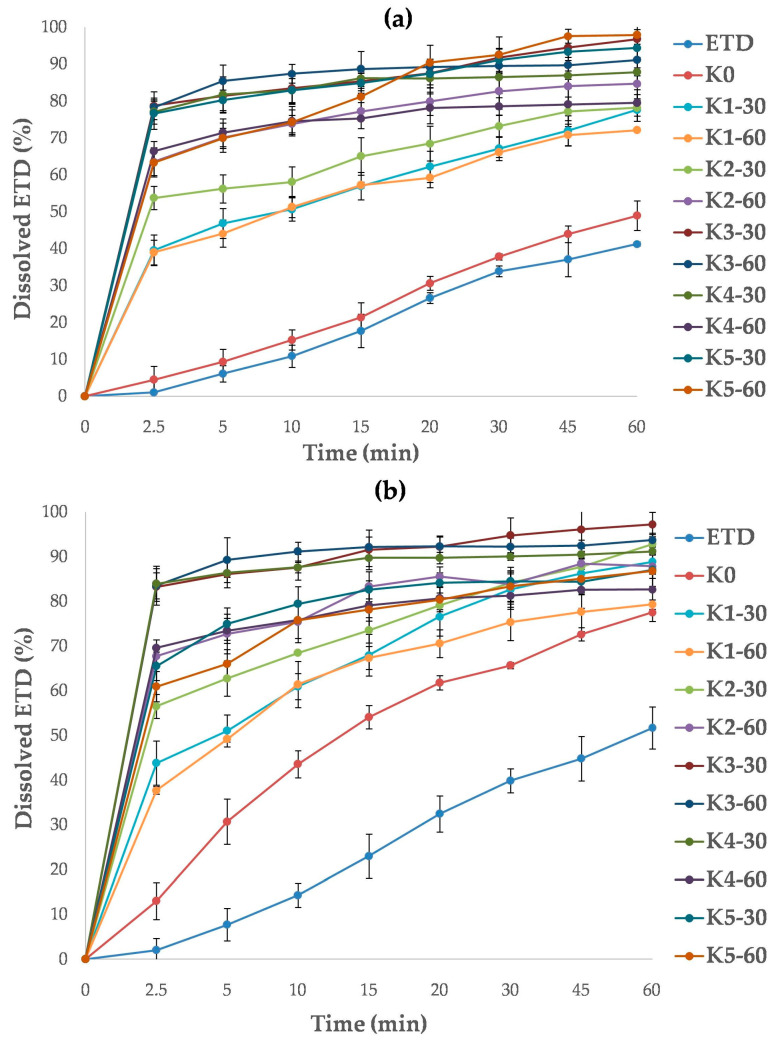
Dissolution profiles of unprocessed ETD, cryo-milled ETD (K0) and ETD from SDs, measured in (**a**) acetate buffer pH 5.5 and (**b**) phosphate buffer pH 7.4.

The dissolution study was performed using two buffers as dissolution media: phosphate buffer (pH 7.4) and acetate buffer (pH 5.5). For all cryo-milled SDs, the dissolution rate of ETD in acetate buffer was significantly improved (*p* < 0.05, Figure 16a). The best dissolution rates were confirmed for K3-30, K3-60, K5-30 and K5-60 formulations and increased from 60.90% to 78.74% at 2.5 min and from 86.76% to 96.74% at 60 min. SDs containing HPMC were characterized by the lowest dissolution rate (ETD dissolution about 80% after 60 min). The ability to improve the dissolution rate can be arranged in the following order: poloxamer > PVP/VA > PVP/VA + poloxamer > HPMC + poloxamer > HPMC. In comparison, the dissolution rate of pure ETD and cryo-milled ETD (K0) was significantly lower, achieving values of 5% at 2.5 min and about 40% at 60 min.

The highest dissolution efficiency of ETD in phosphate buffer was found for K3-30, K3-60 and K4-30 with analyzed values ranging from 77.06% to 78.74% at 2.5 min and from 87.80% to 96.74% at 60 min (Figure 16b). Other SDs reached dissolution rates of about 90% of ETD at 60 min. The lowest dissolution rates were observed for formulations with HPMC (K1-30, K1-60). In contrast, the cryo-milled ETD (K0) revealed a faster dissolution rate of ETD in phosphate buffer than in acetate buffer. This may be a consequence of chemical character of ETD, as it exhibits better solubility in media with higher pH [12]. During the study, no effect of poloxamer concentration and or cryo-milling time (30 or 60 min) on ETD dissolution rate was observed (*p* > 0.05).

**Figure 17:**
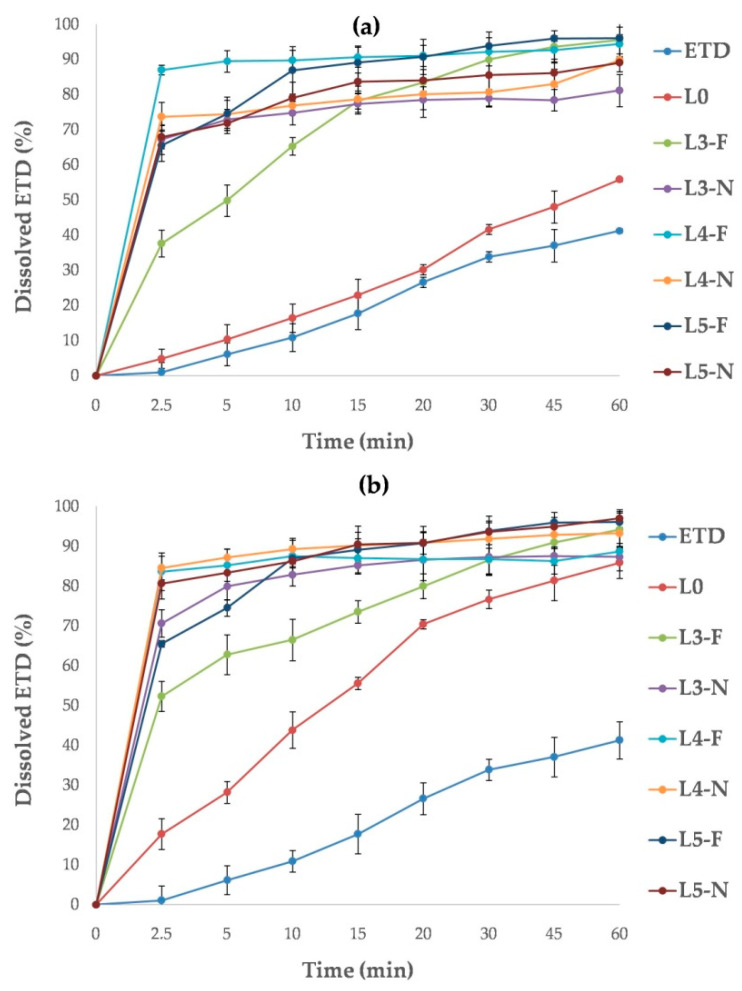
Dissolution profiles of unprocessed ETD, lyophilized ETD (L0) and ETD from SDs measured in (**a**) acetate buffer pH 5.5 and (**b**) phosphate buffer pH 7.4.

The dissolution test for lyophilized SDs demonstrated no impact of poloxamer concentration on the ETD dissolution rate (*p* > 0.05). For each SD, the recorded data were comparable, with dissolution valuesaround90% (L4-N, L5-N) and above 90% (L3-F, L4-F, L5-F) in acetate buffer (Figure 17a), and around90% (L3-N, L4-F) and above 90% (L3-F, L4-N, L5-F, L5-N) in phosphate buffer (Figure 17b). However, the type of freezing (freezer or liquid nitrogen) had a negligible effect on the results obtainedin the study in acetate buffer. The L3-F, L4-F and L5-F formulations (frozen by freezer) exhibited dissolution rates of approximately 95%, whereas theL3-N, L4-N and L5-N formulations (frozen by liquid nitrogen) showed rates ofapproximately87%. This relationship was not confirmed in the test with phosphate buffer. It was surprising that lyophilized ETD (L0, frozen in afreezer) in phosphate buffer provided dissolved 85% of ETD after 60 min, a value similar to that observedfor the SDs.

The formation of soluble complexes between ETD and hydrophilic carriers, as well as the solubilization properties of certain components (e.g., poloxamer), are likely responsible for the improved drug solubility compared to unprocessed ETD [75,76,77]. However, the lower dissolution rate of ETD from cryo-milled SDs containing HPMC may result from the characteristics of the carrier. HPMC is commonly used as a gelling agent to increase the viscosity of formulations [78,79]. It is likely that during the dissolution test, a“gelling” matrix was formed, which delayed the drug’s solubility. The gelling properties of HPMC also hindered the preparation of SDs by lyophilization.

The results of the dissolution test were used to calculate dissolution parameters such as dissolution efficiency (DE) and mean dissolution time (MDT) for all formulations. DE represents the area under the dissolution curve between defined time points, while MDT characterizes the rateof drug dissolution from the formulations after a predetermined time. A higher MDT value suggests stronger holding retention of the drug in the carrier, where as a higher DE value indicates a better dissolution profile [80,81]. All cryo-milled and lyophilized SDs (except K1-30 in acetate buffer) were characterized by MDT values below 10 min (Tables S2 and S3). In comparison, unprocessed ETD, cryo-milled ETD (K0) at both buffer and lyophilized ETD (L0) in acetate buffer had MDT values about 20 min. Only L0 in phosphate buffer had an MDT of about 13 min, which correlated with the results presented in Figure 7. ETD was characterized by minimal DE values of about 0.3% in both buffers, while SDs showed DE values in the range between0.61 and 0.91.

In summary, the dissolution study demonstrated that poloxamer concentration and processing parameters (milling time and freezing method) had no significant effect on the properties of SDs. Furthermore, the results confirmed that HPMC is not an optimal carrier for SDs, as technological difficulties during the lyophilization process and poor dissolution capacity were reported.

The lyophilization method led to greater improvement of ETD solubility compared to cryo-milling due to more efficient molecular dispersion of ETD within the polymer matrix and the presence of the drug mostly in an amorphous form. In the first step of lyophilization, the drug and polymer were dissolved in suitable solvents and then rapidly frozen, which probably stabilized the molecules in their amorphous state during solvent evaporation. Furthermore, the lyophilization method can promote intermolecular interactions, such as hydrogen bonding, that inhibit recrystallization and stabilize the amorphous form. In contrast, cryo-milling primarily reduces particle size and may disrupt the crystalline structure but does not always ensure sufficient molecular dispersion or extensive drug–polymer interactions. Additionally, the porous structure formed during lyophilization increases the surface area of particles and facilitates drug dissolution [37,82,83,84]. These factors explain the more significant improvement in ETD solubility in SDs obtained by lyophilization. Supporting evidence has been reported by Fitriani et al. [84], who characterized solid dispersion with piperine and demonstrated that solid dispersion prepared by lyophilization showed higher solubility due to the porous structure generated during this process, as observed in SEM analysis. Colombo et al. [85] showed that the reduced degree of crystallinity achieved by lyophilization significantly improved solubility, whereas the smallest particle size obtained by milling did not always result in the highest solubility. Verma et al. [86] also observed that the lyophilization process used to obtain solid dispersion with lovastatin enhanced both solubility and dissolution rate. FTIR and DSC analyses confirmed interactions between the drug and carrier and a reduction in the crystalline form of the drug.

## 4. Conclusions

Solid dispersions (SDs) with ETD were developed using two approaches: cryo-milling and lyophilization. Comprehensive analyses, including solubility studies, thermal behavior assessment and morphological characterization, confirmed the suitability of these techniques for optimizing the ETD–SD system. All SD formulations exhibited improved solubility and dissolution rates of ETD in comparison to unprocessed ETD. The formulations obtained via lyophilization and composed of PVP/VA and/or poloxamer as carriers showed the most favorable properties, indicating that these excipients are well suited for this delivery system. These formulations not only enhanced the solubility and dissolution of ETD in aqueous media but also effectively reduced ETD crystallinity.

The data indicate that SDs based on PVP/VA and poloxamer offer a favorable strategy to stabilize ETD in an amorphous state and reduce recrystallization under thermal stress. Consequently, the optimized ETD–SD formulation constitutes a viable platform to incorporate into advanced topical drug delivery systems.

## Figures and Tables

**Table 1:** Methods for SDs characterization [1,26,43].

Type of Method	Method	Examined Feature
Physical examination	Differential scanning calorimetry (DSC), powder X-ray diffraction (XRD), hot stage microscopy (HSM)	Physical state of drug (crystalline or amorphous), degree of drug and carriers’ crystallinity
Surface evaluation	Scanning electron microscopy (SEM), HSM, polarized light optical microscopy	Shape, surface and particle size
Structure explanation	Fourier transform infrared spectroscopy (FTIR), solid state nuclear magnetic resonance spectroscopy (ssNMR)	Type of bonds between drug and carrier
Drug-carrier interactions	DSC, FTIR, NMR	Chemical and physical interactions between drug and carrier
Solubility, dissolution rate	Solubility studies (e.g., shake-flask method), dissolution studies	Rate and extent of drug release

**Table 2:** Composition of ETD-SDs obtained by cryo-milling and lyophilization.

Components	Quantitative Composition (%)	Cryo-Milled Formulation *	Lyophilized Formulation *
ETD	100	K0 (30) K00 (60)	L0 (F) L00 (N)
ETD, HPMC	33.34 + 66.66	K1-30 (30) K1-60 (60)	L1-F (F) L1-N (N)
ETD, HPMC, poloxamer	33.33 + 33.33 + 33.33	K2-30 (30) K2-60 (60)	L2-F (F) L2-N (N)
ETD, PVP/VA	33.34 + 66.66	K3-30 (30) K3-60 (60)	L3-F (F) L3-N (N)
ETD, PVP/VA, poloxamer	33.33 + 33.33 + 33.33	K4-30 (30) K4-60 (60)	L4-F (F) L4-N (N)
ETD, poloxamer	33.34 + 66.64	K5-30 (30) K5-60 (60)	L5-F (F) L5-N (N)

* In brackets, the information about milling time (30 or 60 min) and way of freezing (F—freezer; N—liquid nitrogen) was added.

**Table 3:** The solubility of ETD in the cryo-milled SDs.

Formulation	Solubility (mg/mL)		
	Water	Phosphate Buffer pH 7.4	Acetate Buffer pH 5.5
**ETD**	0.06 ± 0.00	1.59 ± 0.01	0.37 ± 0.02
**K0**	0.08 ± 0.01	1.97 ± 0.05	0.36 ± 0.00
**K00**	0.09 ± 0.01	1.99 ± 0.08	0.37 ± 0.00
**K1-30**	0.12 ± 0.02	2.07 ± 0.18	1.01 ± 0.06
**K1-60**	0.11 ± 0.01	1.98 ± 0.09	0.94 ± 0.03
**K2-30**	0.11 ± 0.01	2.16 ± 0.25	1.31 ± 0.03
**K2-60**	0.16 ± 0.01	2.25 ± 0.18	1.37 ± 0.05
**K3-30**	0.13 ± 0.01	2.04 ± 0.14	0.87 ± 0.06
**K3-60**	0.13 ± 0.01	1.91 ± 0.15	1.10 ± 0.15
**K4-30**	0.11 ± 0.01	2.13 ± 0.12	0.92 ± 0.11
**K4-60**	0.10 ± 0.01	2.38 ± 0.05	0.89 ± 0.03
**K5-30**	0.10 ± 0.01	2.31 ± 0.27	1.03 ± 0.05
**K5-60**	0.13 ± 0.04	2.44 ± 0.16	1.12 ± 0.09

**Table 4:** The solubility of ETD in the lyophillized SDs.

Formulation	Solubility (mg/mL)		
	Water	Phosphate Buffer pH 7.4	Acetate Buffer pH 5.5
**ETD**	0.06 ± 0.00	1.59 ± 0.01	0.37 ± 0.02
**L0**	0.08 ± 0.01	1.60 ± 0.02	0.38 ± 0.00
**L00**	0.09 ± 0.00	1.58 ± 0.02	0.39 ± 0.00
**L3-F**	0.37 ± 0.01	3.08 ± 0.12	1.69 ± 0.16
**L3-N**	0.38 ± 0.01	2.81 ± 0.08	1.59 ± 0.14
**L4-F**	0.43 ± 0.01	2.95 ± 0.45	1.43 ± 0.09
**L4-N**	0.30 ± 0.01	2.18 ± 0.15	1.25 ± 0.23
**L5-F**	0.20 ± 0.01	2.10 ± 0.19	1.22 ± 0.10
**L5-N**	0.26 ± 0.02	2.24 ± 0.03	1.02 ± 0.15

## Data Availability

The original contributions presented in this study are included in the article/Supplementary Material. Further inquiries can be directed to the corresponding author(s).

## Supplementary Materials

The following supporting information can be downloaded at https://www.mdpi.com/article/10.3390/pharmaceutics17111379/s1. Table S1: Composition of PMs; Figure S1: DSC thermogram of PVP/VA (presents the water loss upon heating); Table S2: Measured responses of the dissolution parameters for unprocessed ETD, cryo-milled ETD and cryo-milled SDs; Table S3: Measured responses of the dissolution parameters for unprocessed ETD, lyophilized ETD and lyophilized SDs.
