# Supplementary material for: How Do Cryo-Milling and Lyophilization Affect the Properties of Solid Dispersions with Etodolac?
Source: Pharmaceutics. 2025 Oct 24;17(11):1379. doi: 10.3390/pharmaceutics17111379 (PMC12655227; doi:10.3390/pharmaceutics17111379)
Supplement: Supplementary file 1 [file pharmaceutics-17-01379-s001.zip › pharmaceutics-3869458-supplementary.pdf]

Supplementary Materials

Table S1. Composition of PMs.

PMs	Components	Quantitative composition (%)
PM1	ETD, HPMC	33.34 + 66.66
PM2	ETD, HPMC, poloxamer	33.33 + 33.33 + 33.33
PM3	ETD, PVP/VA	33.34 + 66.66
PM4	ETD, PVP/VA, poloxamer	33.33 + 33.33 + 33.33
PM5	ETD, poloxamer	33.34 + 66.66

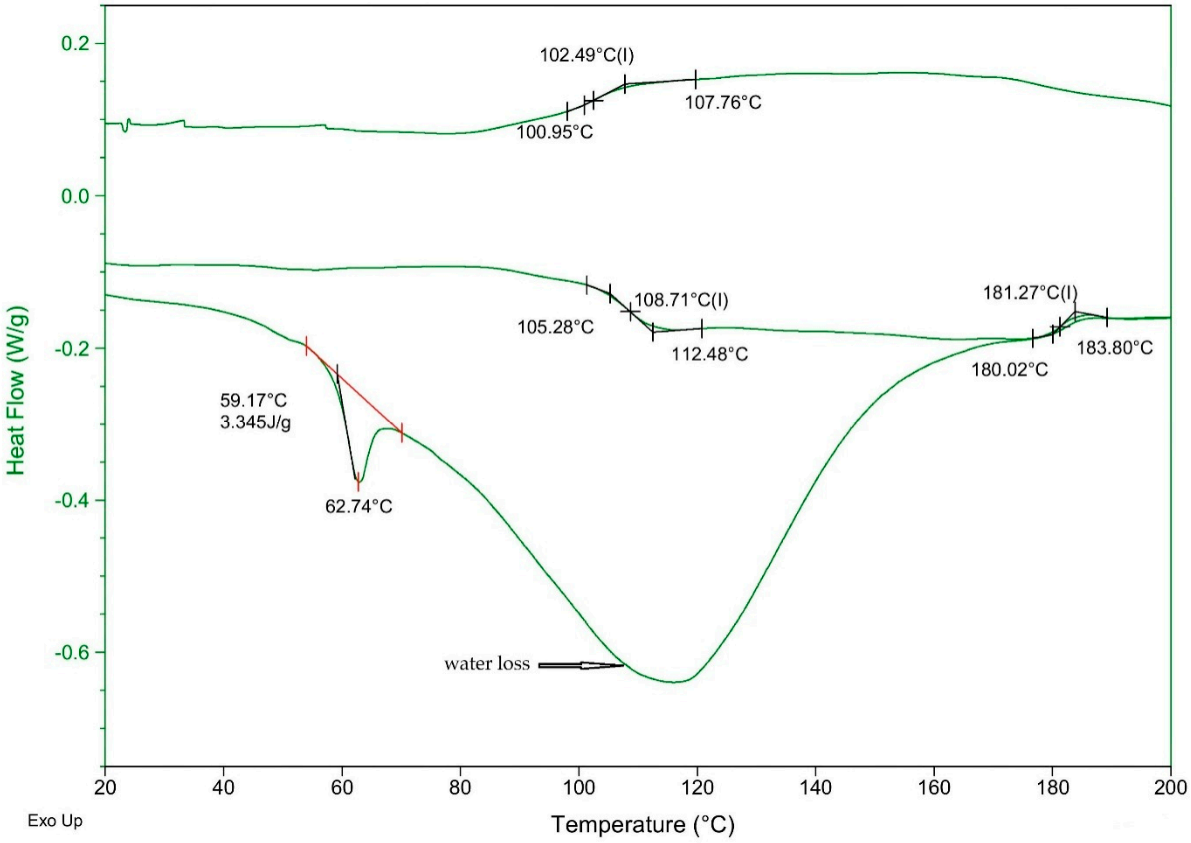

Figure S1. DSC thermogram of PVP/VA (presents the water loss upon heating).

Table S2. Measured responses of the dissolution parameters for unprocessed ETD, cryo-milled ETD and cryo-milled SDs.

Formulation	Measured responses			
	DE (%)		MDT (min)	
	pH 5.5	pH 7.4	pH 5.5	pH 7.4
ETD	0.28	0.34	19.87	20.88
K0	0.33	0.60	20.02	19.35
K1-30	0.63	0.75	11.29	9.20
K1-60	0.61	0.69	8.99	7.63
K2-30	0.69	0.79	7.13	8.88
K2-60	0.78	0.82	4.44	4.11
K3-30	0.88	0.91	5.31	3.69
K3-60	0.87	0.90	2.79	2.42
K4-30	0.84	0.88	2.67	2.31
K4-60	0.76	0.78	2.97	3.11
K5-30	0.87	0.81	4.53	4.16
K5-60	0.87	0.79	6.56	5.36

Table S3. Measured responses of the dissolution parameters for unprocessed ETD, lyophilized ETD and lyophilized SDs.

Formulation	Measured responses			
	DE (%)		MDT (min)	
	pH 5.5	pH 7.4	pH 5.5	pH 7.4
ETD	0.28	0.34	19.87	20.88
L0	0.35	0.67	21.76	13.45
L3-F	0.81	0.80	9.04	8.83
L3-N	0.76	0.84	3.80	2.46
L4-F	0.90	0.85	2.92	2.55
L4-N	0.79	0.89	6.93	2.55
L5-F	0.89	0.89	4.45	2.30
L5-N	0.82	0.90	4.85	4.32
